# Association of ACL Laxity Tests with Arthroscopic Findings in Chronic ACL and PCL Deficient Knees

**DOI:** 10.1055/s-0038-1637008

**Published:** 2018-03-26

**Authors:** S. Sazali, A. Rusdi, H. T. Siti

**Affiliations:** 1 Department of Arthroscopy and Sports Injury, Hospital Kuala Lumpur Malaysia, Kuala Lumpur, Malaysia

**Keywords:** chronic ACL and PCL injury, ACL laxity tests, association between ACL laxity tests and arthroscopy findings

## Abstract

An anterior cruciate ligament (ACL) injury may be diagnosed by clinical examination and radiological investigation using magnetic resonance imaging or by arthroscopy. ^1,2^ Based on our experience, the ACL tear in concomitant chronic ACL and posterior cruciate ligament (PCL) deficient knees may produce knee laxity, which is more difficult to assess on clinical examination, which in turn may affect the management algorithm of the patient. Our hypothesis is that, in a concomitant chronic ACL and PCL injury, posterior capsular contracture and abnormal reattachment of torn ACL will result in less clinical and subjective laxity, preoperatively. The aim of this study is to review a cohort of patients who had undergone PCL reconstructive surgery and compare the preoperative clinical assessments with and without anesthesia with arthroscopic finding of ACL. This is to assess the accuracy and reliability of clinical ACL laxity tests in detecting ACL tear in chronic ACL and PCL injury.

An anterior cruciate ligament (ACL) injury may be diagnosed by clinical examination and radiological investigation using magnetic resonance imaging (MRI) or by arthroscopy. 1 2 Based on our experience, the ACL tear in concomitant chronic ACL and posterior cruciate ligament (PCL) deficient knees may produce knee laxity, which is more difficult to assess on clinical examination, which in turn may affect the management algorithm of the patient. Our hypothesis is that, in a concomitant chronic ACL and PCL injury, posterior capsular contracture and abnormal reattachment of torn ACL will result in less clinical and subjective laxity, preoperatively. The aim of this study is to review a cohort of patients who had undergone PCL reconstructive surgery and compare the preoperative clinical assessments with and without anesthesia with arthroscopic finding of ACL.

## Methodology

This study was a retrospective analysis of all 38 patients with chronic PCL tear (> 6 weeks post injury), who underwent PCL reconstruction in 2012 and 2013 in our center. In all the cases, the clinical diagnosis of chronic PCL with or without ACL injury was made after examination by the senior author through three clinical examinations comprising (1) Lachman test, 3 4 recording the degree of anterior laxity (normal, 3–5mm, 6–10 mm or > 10 mm) and the quality of the end point (firm, intermediate, or absent); (2) anterior drawer test 3 4 and (3) pivot shift test, 3 4 and an optional MRI scan, when there was diagnostic uncertainty. Preoperative clinical function was assessed using the International Knee Documentation Committee Subjective Knee Evaluation Form 5 within 2 days prior to surgery. At the time of surgery, the same three clinical examinations were performed under anesthesia by the senior author. No arthrometry was performed. Intraoperatively, the arthroscopic appearance of the ACL was classified as intact, partial, or complete tear. 6

## Results

**Fig. 1:**
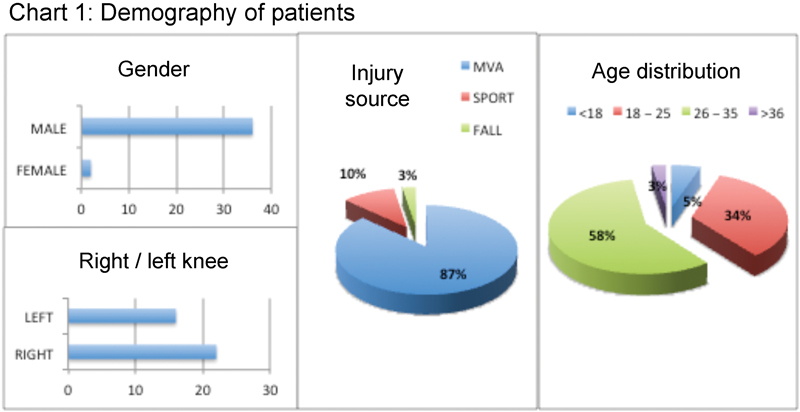
Demography of patients. Abbreviation: MVA, motor vehicle accident.

**Fig. 2:**
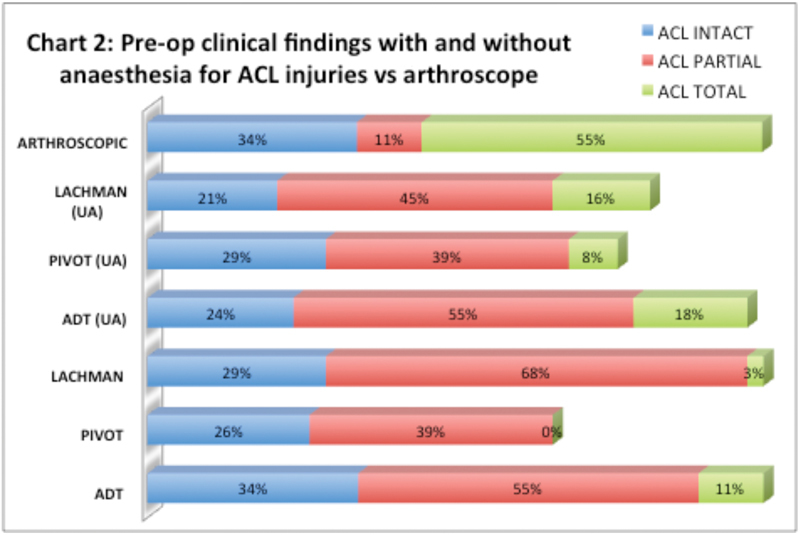
Preoperative clinical findings with and without anesthesia for ACL injuries versus arthroscope. Abbreviations: ACL, anterior cruciate ligament; ADT, anterior drawer test; UA, under anesthesia.

**Fig. 3:**
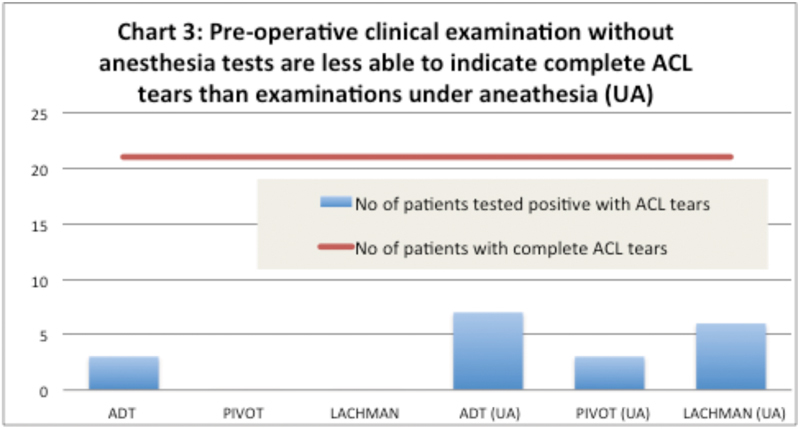
Preoperative clinical examination without anesthesia tests is less able to indicate complete ACL tears than examinations UA. Abbreviations: ACL, anterior cruciate ligament; ADT, anterior drawer test; UA, under anesthesia.

Data was collected from 38 patients who were diagnosed and underwent PCL reconstruction surgery to the knee. Seventeen cases had ACL and PCL injury, while 21 cases had combined multiligament injuries involving ACL, PCL, and collateral ligaments. The cohort of patients reviewed consisted mainly of males, aged between 26 and 35, and injuries were mainly due to motor vehicle accidents ( Fig. 1 ). Of the 38 patients who underwent surgery, 25 (66%) patients suffered from ACL injuries, of which 23 (55%) suffered from total ACL tear, whereas 2 (11%) suffered from partial ACL tear ( Fig. 2 ). Based on the data collected, all clinical examinations were found to be highly inaccurate with disparity ranging between 67% and 100% as compared with arthroscopy findings ( Fig. 3 ).Comparatively, clinical examinations performed under anesthesia were better to indicate ACL tears than preoperative tests, with anterior drawer test 7(33%) and Lachman 6(29%) tests providing significantly better results than preoperative tests without anesthesia ( Fig. 3 ).

## Discussion

We found that in chronic PCL injury knees the accuracy of preoperative clinical examination for ACL laxity with or without anesthesia is low. Although preoperative examination under anesthesia is more sensitive compared with the preoperative examination without anesthesia, it was still less accurate based on the intraoperative arthroscopic findings. Preoperative test without anesthesia was virtually ineffective in indicating ACL tears in patients with ACL tear, with two of three clinical examinations yielding no indication of ACL tears in any patient. This may be due to the posterior capsular contracture in chronic PCL injury or an abnormal reattachment of torn ACL. Muscle relaxation during the examination under anesthesia might also influence the findings. We believe that our findings should be borne in mind when examining a knee with chronic PCL injury of a possible concomitant ACL injury.

## Conclusion

In chronic PCL injury, preoperative examination with or without anesthesia alone may give an inaccurate finding to determine the diagnosis and grading of ACL laxity. The diagnosis and management of ACL injuries should be based on the history, clinical examination, and arthroscopic findings. MRI should be interpreted with caution as the reattached ligament may be confused with a partially injured or uninjured ligament.
